# Supplementary material for: Asciminib Mitigates DNA Damage Stress Signaling Induced by Cyclophosphamide in the Ovary
Source: Int J Mol Sci. 2021 Jan 30;22(3):1395. doi: 10.3390/ijms22031395 (PMC7866503; doi:10.3390/ijms22031395)

Supplementary figures.

Supplementary Figure 1 Asciminib attenuates DNA Damage Response induced by cyclophosphamide. (A) Western blotting analysis and relative quantification of ovarian lysates from female pups (p7) injected with Cy 100mg/kg alone or in combination with various doses of Asciminib as indicated and sacrificed within 18 h after injection. Cy injection did induce the phosphorylation on H2AX on Ser139 (γH2AX) *in vivo* in mice. Of note, γH2AX was absent in the controls and partially prevented by co-treatment with Asciminib.

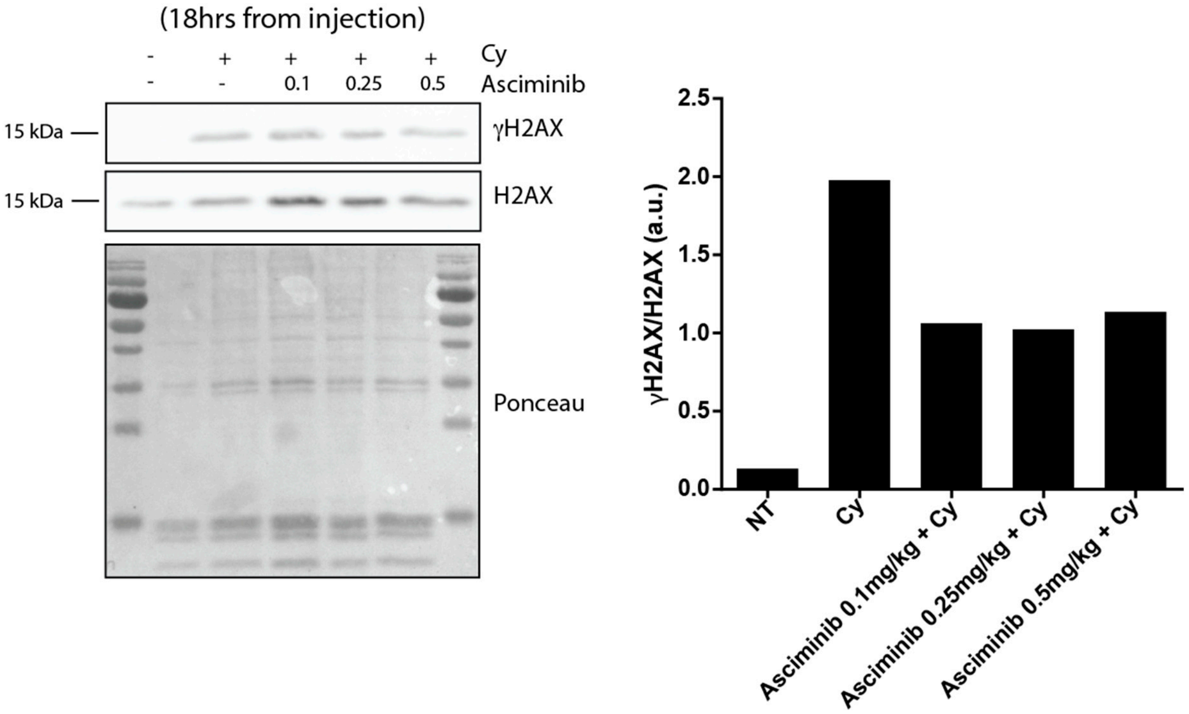

Supplementary Figure 2. A high dose of Asciminib did not protect the ovarian reserve from cyclophosphamide treatment. Ovaries of each experimental group were dissected three days after injection (mice were injected using with Cy 100 mg/kg alone or in tandem with Asciminib 1 mg/kg**)** and then analyzed by IHC assay with Msy2 antibody. Ovaries from independent experiments were analyzed. In the box plot, each dot represents the average primordial + primary follicles numbers per section of each gonad analyzed. Scale Bar 100μm.

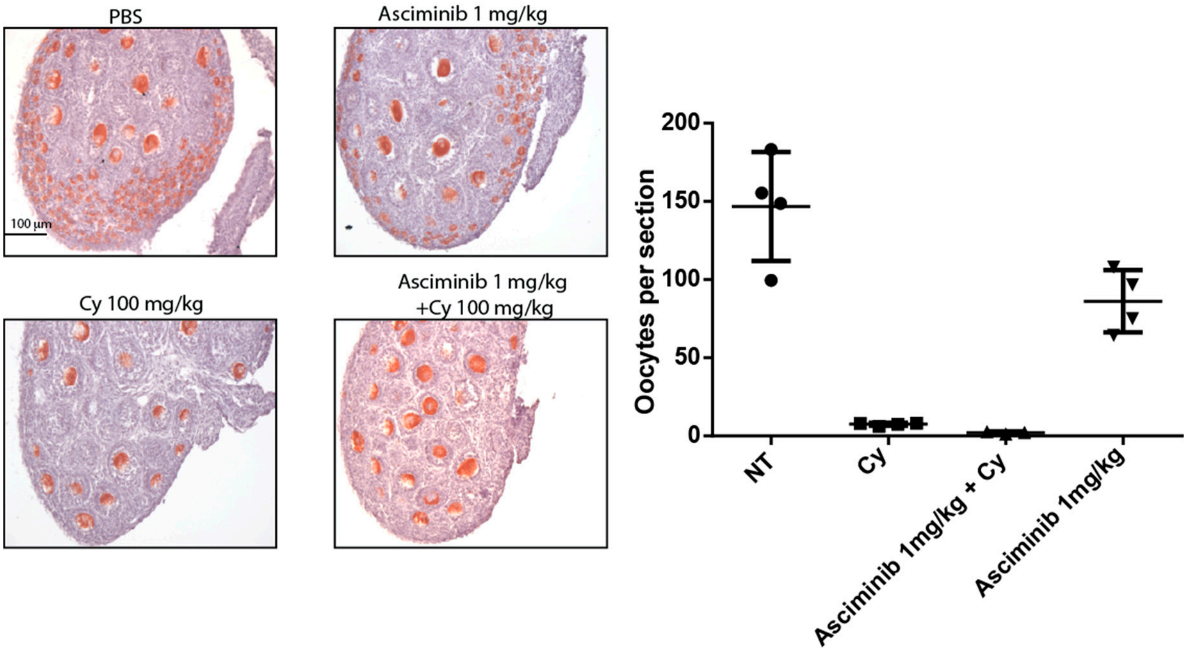

List of various Antibodies used in our IF assays

Antigen	Host	Function	Purchased from
Msy-2	Mouse	Germ cells- specific protein (cytosolic)	Santa Cruz (sc-21316)
p-AKT (T308)	Rabbit	Follicle activation pathway	Santa Cruz sc-16646-R
p63	Rabbit	Germ cell- specific protein (nuclear)	Home-made
γH2AX (S139)	Mouse	DNA damage early marker	Millipore 05-636
p-DNA-PK (S2056)	Rabbit	DDR apical kinase	SAB4504169 Sigma Aldrich
p-ATM (S1981)	Mouse	DDR apical kinase	Rockland 200-301-500
p-p53 (S15)	Rabbit	DDR effector	Cell Signaling Technology (9284)
Cleaved PARP	Rabbit	Nuclear Marker for Apoptosis	Cell Signaling Technology (9544)

Schematic representation of the treatment and the timing of ovary collection.

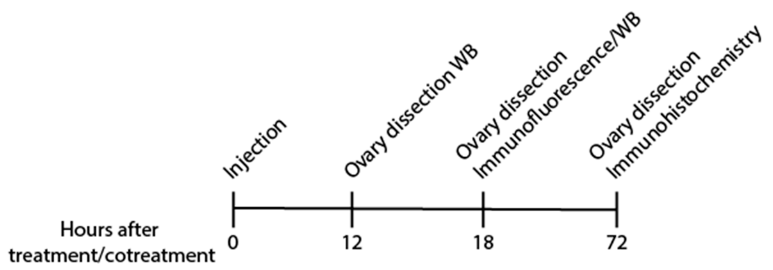

Supplement: Supplementary file 1 [file ijms-22-01395-s001.pdf]
